# Eleven-Week Preparation Involving Polarized Intensity Distribution Is Not Superior to Pyramidal Distribution in National Elite Rowers

**DOI:** 10.3389/fphys.2017.00515

**Published:** 2017-08-02

**Authors:** Gunnar Treff, Kay Winkert, Mahdi Sareban, Jürgen M. Steinacker, Martin Becker, Billy Sperlich

**Affiliations:** ^1^Division of Sports and Rehabilitation Medicine, Ulm University Hospital Ulm, Germany; ^2^Institute of Sports Medicine, Prevention and Rehabilitation, Paracelsus Medical University Salzburg, Austria; ^3^Data Mining and Information Retrieval Group, Julius-Maximilian University Würzburg, Germany; ^4^Integrative and Experimental Training Science, Institute of Sport Science, University of Würzburg Würzburg, Germany

**Keywords:** rowing, training intensity distribution, elite athletes, interval training, high intensity, high volume, training zones

## Abstract

Polarized (POL) training intensity distribution (TID) emphasizes high-volume low-intensity exercise in zone (Z)1 (< first lactate threshold) with a greater proportion of high-intensity Z3 (>second lactate threshold) compared to Z2 (between first and second lactate threshold). In highly trained rowers there is a lack of prospective controlled evidence whether POL is superior to pyramidal (PYR; i.e., greater volume in Z1 vs. Z2 vs. Z3) TID. The aim of the study was to compare the effect of POL vs. PYR TID in rowers during an 11-wk preparation period. Fourteen national elite male rowers participated (age: 20 ± 2 years, maximal oxygen uptake ($\dot{\text{V}}$O_2max_): 66 ± 5 mL/min/kg). The sample was split into PYR and POL by varying the percentage spent in Z2 and Z3 while Z1 was clamped to ~93% and matched for total and rowing volume. Actual TIDs were based on time within heart rate zones (Z1 and Z2) and duration of Z3-intervals. The main outcome variables were average power in 2,000 m ergometer-test (P_2,000 m_), power associated with 4 mmol/L [blood lactate] (P_4[BLa]_), and $\dot{\text{V}}$O_2max_. To quantify the level of polarization, we calculated a Polarization-Index as log (%Z1 × %Z3 / %Z2). PYR and POL did not significantly differ regarding rowing or total volume, but POL had a higher percentage of Z3 intensities (6 ± 3 vs. 2 ± 1%; *p* < 0.005) while Z2 was lower (1 ± 1 vs. 3 ± 2%; *p* < 0.05) and Z1 was similar (94 ± 3 vs. 93 ± 2%, *p* = 0.37). Consequently, Polarization-Index was significantly higher in POL (3.0 ± 0.7 vs. 1.9 ± 0.4 a.u.; *p* < 0.01). P_2,000 m_ did not significantly change with PYR (1.5 ± 1.7%, *p* = 0.06) nor POL (1.5 ± 2.6%, *p* = 0.26). $\dot{\text{V}}\text{O}$_2max_ did not change (1.7 ± 5.6%, *p* = 0.52 or 0.6 ± 2.6, *p* = 0.67) and a small increase in P_4[BLa]_ was observed in PYR only (1.9 ± 4.8%, *p* = 0.37 or −0.5 ± 4.1%, *p* = 0.77). Changes from pre to post were not significantly different between groups in any performance measure. POL did not prove to be superior to PYR, possibly due to the high and very similar percentage of Z1 in this study.

## Introduction

Olympic rowers aim to cover the distance of 2,000 m faster than their opponents, with world best times varying from 5:18 to 7:08 min. For this purpose, rowers generate an average power of ~590 W (Voliantis and Secher, 2009) and exhibit an outstanding maximal oxygen uptake ($\dot{\text{V}}$O_2max_; Secher, 1993; Ingham et al., 2002) with extreme acidosis and metabolic stress (Nielsen, 1999).

To prepare for this kind of physical exertion and performance, elite rowers perform most of their training time with high-volume and (relatively) low-intensity exercise (Nybo et al., 2014), often referred to as “basic endurance training,” which is quite demanding in rowing as indicated by an oxygen uptake exceeding 3.5 L/min (Secher, 1993) and forces >500 N per stroke (Roth et al., 1993). The high metabolic and muscular demands limit high-volume low-intensity rowing sessions to 90–100 min to avoid impaired stroke technique. With increasing boat velocity the energy expenditure increases 2.2- to 2.4-fold (Secher, 1993) ultimately forcing the athlete to row as efficiently as possible during each session. In addition to the rowing sessions, most rowers implement 2–3 strength-training sessions per week into their training schedules, thereby increasing the exhaustive character of a rower's preparation for competition.

Over the last three decades the total training volume in elite rowing elevated by 66%, augmenting to ~23 h/wk in Norwegian rowers during the 90's (Fiskerstrand and Seiler, 2004) and is estimated to peak at ~29 h/wk nowadays (Nielsen, 2009/2017). Based on temporal limits and high workload of today's elite rowers we may assume that total training volume is near its functional maximum. As the medal winners' 2,000 m boat speed still increases by ~0.12% per year (Kleshnev and Nolte, 2011), optimization of training schedules, especially by altering the intensity distribution (TID) might be a worthwhile resource to enhance performance.

Within the literature a three-zone intensity model is applied to quantify the TID. The model is based on the following physiological benchmarks: Zone 1 (Z1) is defined as low-intensity exercise with low levels of blood lactate below first lactate or ventilatory threshold. Zone 2 (Z2) refers to elevated and accumulated blood lactate concentration also called “lactate threshold training” identified as an intensity between the first and second lactate or ventilatory threshold. Finally, Zone 3 (Z3) refers to high-intensity exercise above the second lactate or ventilatory threshold (Kindermann et al., 1979; Seiler and Kjerland, 2006). Common additional parameters to demarcate the three intensity zones are based on blood lactate levels [BLa] of <2 mmol/L (Z1), 2–4 mmol/L (Z2), and >4 mmol/L (Z3) as well as certain percentages of maximal heart rate or maximal oxygen uptake. Noteworthy, national sport governing bodies and rowing federations often apply five-zone models to differentiate training intensities more detailed (Seiler, 2010).

So far, few studies have described the TID in rowing, especially on an elite level. In 1998 Steinacker et al. reported German, Danish, Dutch, and Norwegian elite rowers spending 90–96% of their total training volume in Z1 but it remains uncertain to the reader how the remaining percentage was exactly split into Z2 and Z3 (Steinacker et al., 1998). Single case studies reported a TID of 85% in Z1 (Nybo et al., 2014) in an elite lightweight rower from Denmark and ~81% in Z1 in a double Olympic champion from Norway (Seiler and Tønnessen, 2009), with both reports not specifying the percentages spent in Z2 and Z3. Most analyses so far report a pyramidal TID, i.e., decreasing amount of training spent in Z1, Z2 and Z3. German junior rowers e.g., exhibited a TID of ~95-3-2 (i.e., percentage in Z1, Z2, Z3) during the last 9 weeks before the first competition (Guellich et al., 2009) and nine successful Olympic rowers from New Zealand featured a TID of 77-17-6 (Plews et al., 2014). A successful French rower employed a TID of 45% Z1 and 55% Z2 (Lacour et al., 2009), notably a TID emphasizing “threshold” intensity. Only one investigation so far reported a polarized (POL) TID of 93-2-5 in a Belgian elite sculler (Bourgois et al., 2013). POL is characterized by a relatively high amount of volume performed in Z1 and Z3, with less volume in Z2. Taking into account that rowing is a high-intensity sport and being aware of several reports from other endurance disciplines like e.g. running, cycling or cross-country skiing (Stöggl and Sperlich, 2015), the long-term stimulus of POL may improve endurance performance with potentially less autonomic and hormonal stress and boredom, which is supported by experiments in club rowers who especially emphasized Z3-training (Driller et al., 2009; Ní Chéilleachair et al., 2016).

Several observational studies of national or world-class athletes from various sport disciplines like running (Billat et al., 2001) or cross-country skiing (Seiler and Kjerland, 2006; Sandbakk et al., 2011; Tønnessen et al., 2014) successfully applied a POL TID. Only one controlled study in 18 club rowers following a 28-day detraining period reported a similar increase of ergometer performance with POL (72-0-28) compared to a control group exaggerating low-intensity rowing (98-2-0; Ingham et al., 2008).

Integrating the findings of rowing studies as well as findings from other endurance sports (Neal et al., 2013; Stöggl and Sperlich, 2014) strong evidence exists that POL may be applied in high performance rowing, but this notion is drawn on two serious limitations: Firstly, performance benefits of POL have been concluded based on retrospective observations, but prospective randomized-controlled data on sub-elite or elite level rowers do not exist. Secondly, POL has been compared to static TIDs which do not change over weeks or months. From a methodological point of view, experiments involving static TIDs are convenient for scientists to compare differences between groups, but a static TID does not mirror real-training scenarios in high performance sports, in which TIDs are shaped “dynamically” with increasing percentages of Z2 and Z3 before competitions. Notably, this is recommended by the current scientific literature (Bangsbo et al., 2010; Tønnessen et al., 2014).

Altogether, numerous successful TIDs exist in rowing (i.e., POL and PYR), data from various disciplines and rowing are conflicting, and no prospective randomized-controlled investigation exists comparing POL to a dynamic TID in elite rowers. Therefore, we aimed to compare 11 weeks of a competition-preparation period involving POL to a dynamic PYR distribution in national elite rowers.

## Materials and methods

### Design

The present prospective study was conducted during the final 11 weeks of the preparation period, i.e., calendar week 3–14. Immediately after the study, the national qualification regatta to apply for the national team was scheduled. Fourteen national elite rowers participated in the study. Twelve (86%) rowed for Germany on international regattas in 2016, three rowers were lightweights. Table 1 summarizes the rowers' anthropometric data. All rowers provided written informed consent to participate. The experimental protocol was approved by the ethical review board of the University of Ulm.

**Table 1:** Participants' anthropometric characteristics.

**Variable**	**PYR**	**POL**	***p***	***d*_Cohen_**
Standing height (cm)	193 ± 2	185 ± 7	0.029	−1.55
Body mass (kg)	93 ± 3	85 ± 11	0.138	−0.99
Age (years)	19 ± 1	21 ± 2	0.062	0.26
$\dot{\text{V}}$O_2max_ (mL/min/kg)	64 ± 3	68 ± 7	0.171	0.74

*PYR (n = 7), pyramidal training intensity distribution; POL (n = 7), polarized training intensity distribution; $\dot{\text{V}}$O_2max_, maximal oxygen uptake*.

The rowers trained in two different training facilities (A;B) within Germany. Athletes could not be randomly assigned to PYR or POL, because several rowers trained in crew boats. Moreover, it was not possible to separate existing training squads for organizational reasons. To overcome this limitation, we allocated two groups in each of the two facilities to either PYR or POL. In each facility, one training group followed the traditional rowing schedule emphasizing high-volume low-intensity exercise and a pyramidal TID (PYR). The other group targeted a polarized TID model (POL). In facility A, one athlete of each group was excluded from the study due to illness or injury not related to the intervention. The 11-wk duration included pre- and post-test procedures to evaluate the changes in rowing performance and physiological variables.

### Training intervention

#### Training intensity zones

A three-zone training model was applied to quantify TID (Foster et al., 2001; Seiler and Kjerland, 2006; Seiler, 2010). The following three intensity zones were established based on a 5 × 4-min ergometer step test as described in detail below (Section Power Output at 2 and 4 mmol/L Blood Lactate): Z1 was defined as the intensity between 65% of maximal heart rate and the first lactate threshold or lactate-equivalent as described by Kindermann et al. (1979). Z2 was defined as the intensity between first lactate threshold and the second or individual lactate threshold as described by Dickhuth et al. (1991). Z3 was defined as an intensity above the second lactate threshold.

All intensities were related to the corresponding heart rates (HR) during the ergometer step test to allow for objective entries into the mandatory online training diary of the German Rowing Federation. The diary included information about the training mode, duration, distance, and intensity as well as information on days off and illness or injury. Entries of Z1 and Z2 sessions were based on time spent in corresponding HR-zones. HR was measured by the athletes' own HR-monitors and/or with the smartphone based rowing in Motion–App (In Motion Software & Sports Technology, Hanau, Germany) that was connected to a chest belt with Bluetooth data transmission (H7, Polar Electro, Oy, Finland). To avoid underestimation of Z3 sessions due to the delayed HR-response at high intensities, Z3 sessions were not documented by time in corresponding HR-zone, but by the total duration of the performed Z3-interval, as long as the maximal HR of the interval reached the individually defined Z3 HR-zone. Otherwise the interval was rated as Z2.

The diary logs were checked by the coaches and crosschecked by the research team for plausibility. After completion of the study, all data were exported (.csv files) and subsequently analyzed using the Python data analysis toolkit “pandas” (version 0.18.0, PyData Development Team) and the Scientific Computing Library “Scipy” (version 0.17.0, SciPy developers).

Notably, a basic framework of adequate training intensities was provided by rowing stroke frequency and pace prescribed by the coaches, which is a common practice in rowing (Plews et al., 2014).

#### Training modes

Training differentiated four modes, namely (i) *Rowing*: involving boat and ergometer rowing, (ii) *Endurance*: other endurance training like running, cycling, swimming, etc., (iii) *Strength*: resistance training, machine-based or weight lifting, and (iv) *Other*: stretching, stability training, etc.

#### Training intensity distribution

The overall training of both groups included all four training modes (*Rowing, Endurance, Strength*, and *Other*). Based on the coaches' experience with their athletes, both groups targeted 16–18 h total training volume per week and ~120 km of rowing per week. The general training schedule provided 2–3 sessions of strength training and 6–8 rowing sessions per week. The primary distinction between the two groups was the prescribed intensity distribution, with PYR including two to three Z2-sessions (e.g. 1 × 4 or 2 × 3 or 3 × 2 km) with not more than one session in Z3. In contrast, POL included 2-3 sessions of Z3 training (e.g. 2 × 2 km, 10 × 250 m; 6 × 1 km) while avoiding Z2 as much as possible.

To ensure high compliance, the general training prescription was discussed with the coaches of both groups before initiating the study. However, since preparation period is fundamental for competition success, the coaches were permitted to adapt the schedule depending on the athletes' particular needs, health status and environmental conditions.

### Pre- and post-measurements

During the first visit all rowers were medically examined by a licensed sports physician. The medical examination also included an electrocardiography at rest (CardioPart 12 Blue, Amedtec, Aue, Germany), an echocardiogram (Philips CX50, Phillips Medical Systems, Andover, MA, USA), and blood analysis to exclude iron deficiency and anemia. All rowers were declared free from cardiovascular disease and eligible to perform the exercise protocol and the study.

**Figure 1:**
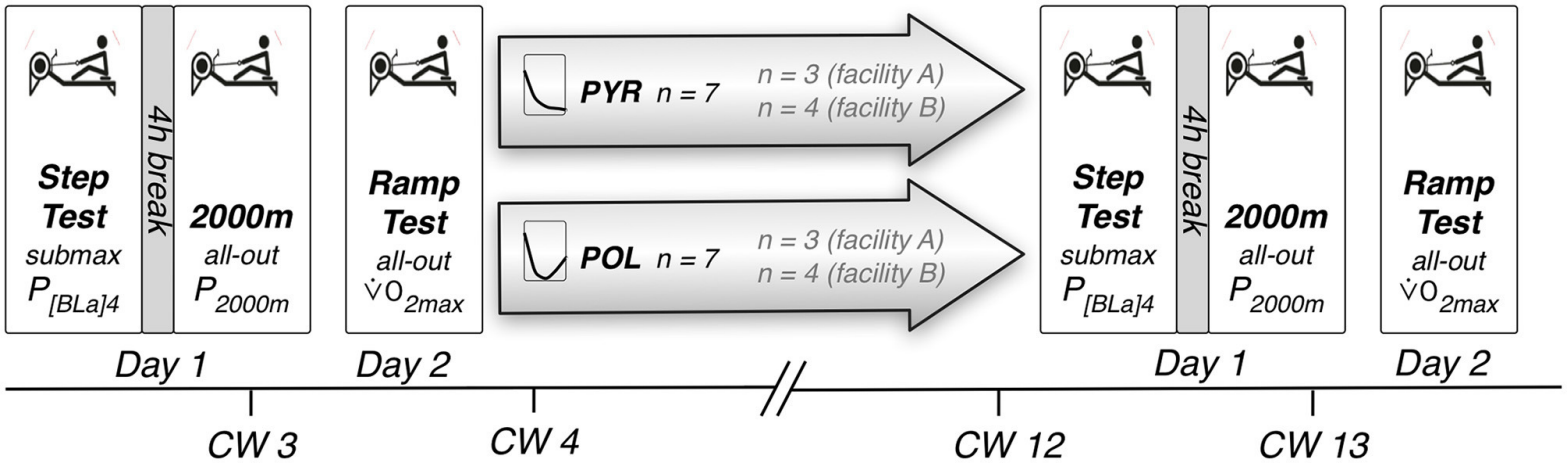
Study and test design. P_[BLa]4_, Power at blood lactate concentration of 4 mmol/L; P_2,000 m_, Average power in 2,000 m rowing ergometer test; $\dot{\text{V}}$O_2max_, Maximal oxygen uptake; PYR, Pyramidal training intensity distribution; POL, Polarized training intensity distribution; CW, Calendar week.

Afterwards, a series of rowing ergometer tests was conducted on two days (Figure 1) employing a Concept 2 Type D ergometer (Concept 2, Morrisville, USA) for all tests. The ergometer was modified with a load cell for force measurement and a rotary transducer to calculate the power output [Institut für Forschung und Entwicklung von Sportgeräten (FES), Berlin, Germany].

#### Power output at 2 and 4 mmol/L blood lactate

After the physical examination, all rowers performed a 5 × 4-min incremental step test with 50 W increments per stage. The workloads ranged from 150 to 350 W in the lightweight and from 200 to 400 W in the open weight class rowers. During a 30-s break between each stage, 20 μL of capillary blood were sampled from the hyperemic earlobe and the level of blood lactate was immediately analyzed amperometric-enzymatically (C-Line, EKF, Barleben, Germany). A specialized software calculated power output at 2 and 4 mmol/L [blood lactate] (P_2[BLa]_ and P_4[BLa]_) using a polynomic fitting of the power and lactate data (Winlactat, Mesics, Münster, Germany). P_4[BLa]_ is an accepted measure of rowing performance with standard errors of the estimate of 2,000 m ergometer performance amounting to 1.4–3.3% (Smith and Hopkins, 2012). We used 3.3% as the lower limit to identify worthwhile changes, since the performance level of our rowers was similar to those of previous reports (Nevill et al., 2011).

#### Two thousand meters ergometer test

All rowers performed an all-out 2,000 m ergometer test to evaluate maximal rowing ergometer performance, by covering the virtual distance of 2,000 m as fast as possible. The average power (P_2,000 m_) was recorded from the Concept 2 monitor afterwards. This test is employed worldwide in elite rowing to determine changes of maximal performance (Hahn et al., 2000; Mäestu et al., 2005; Smith and Hopkins, 2012). The standard error of the estimate of 2,000 m single-scull performance has been calculated to be 2.6% (Jürimäe et al., 2000). The error of measurement on Concept 2 rowing ergometers for P_2,000 m_ amounts to 1.3% (95%CI 0.9-2-9; Soper and Hume, 2004). We used this value to estimate the smallest worthwhile change in P_2,000 m_ (Smith and Hopkins, 2011).

#### Measurements of maximal oxygen uptake

$\dot{\text{V}}$O_2max_ was measured with a ramp test protocol that enables a linear increase in power and objective test termination (Winkert et al., 2016). Briefly, target power of the rowing ergometer was initially set to 160 W and increased 30 W/min (lightweight rowers) or 35 W/min (open weight class). The test automatically terminated in case the rowers failed to increase power within a 7-W range of five strokes. Gas exchange and ventilation were measured using a metabolic analyzer with a dynamic mixing chamber (Metamax 3x, Cortex Biophysics, Leipzig, Germany). The technical error of measurement of this device amounts to 0.03–0.21 L/min (95% confidence interval; Larsson et al., 2004). The system was calibrated prior to each test using ambient air and the manufacturers' calibration gas (16% O_2_, 5% CO_2_). A precision 3-L syringe (Hans Rudolph, Shawnee, USA) was employed to calibrate the flow sensor.

$\dot{\text{V}}$O_2max_ was defined as the highest $\dot{\text{V}}$O_2_ with increasing workload and averaged over a 30-s interval. $\dot{\text{V}}$O_2max_ was considered when $\dot{\text{V}}$O_2_ failed to increase with progressive work rate (leveling off) or at least a plateau of $\dot{\text{V}}$O_2_ was present. A plateau was defined as an increase in $\dot{\text{V}}$O_2_ <150 mL/min, which is the most frequent definition in literature (Midgley et al., 2007). A leveling-off or plateau $\dot{\text{V}}$O_2_ was found in all cases. In addition, respiratory exchange ratio at exertion was always >1.1 with [BLa] ≥ 8 mmol/L.

#### Polarization-index

To quantify the individual level of periodization, we calculated a Polarization-Index based on the percentage, time, or distance trained in each intensity zone. The Polarization-Index was calculated as follows:

(1) Polarization − Index (a.u.)=log (Z1/Z2 × Z3)

If Zone 2 = 0, following formula avoided zero in the denominator:

(2) Polarization − Index (a.u.)=log (Z1/0.1 × (Z3−0.1))

If Z3 = 0, Polarization-Index was defined as zero.

In case of a Polarization-Index > 2.0 a.u., the TID was defined to be polarized, indicating an increasing level of polarization with higher values. If Polarization-Index was ≤ 2 a.u. the TID was defined as being not polarized.

In the context of this study, all TIDs with a Polarization-Index ≤ 2.0 a.u. were pyramidal or indifferent, but from a theoretical perspective, a Polarization-Index ≤ 2.0 can indicate at least five different TIDs, namely (i) a pyramidal TID (80-15-5; Polarization-Index = 1.4 a.u.), (ii) an inverse pyramidal TID (20-30-50; Polarization-Index = 1.5 a.u.), (iii) a *Lactate Threshold TID* (60-38-2; Polarization-Index = 0.5 a.u.), an indifferent TID (90-5-5; Polarization-Index = 2.0 a.u.), or a Long-Slow-Distance TID (100-0-0; Polarization-Index = 0.0 a.u.).

A Polarization-Index > 2.0 a.u. does not indicate a polarized distribution, if Z1 is smaller than Z3 (e.g., 40-0-60; PI = 4.4 a.u.). This kind of TID would probably be classified as *High Intensity Training* or *HIT*, since polarized distributions necessitate Z1 volume to be highest.

Even if we assume that a given Polarization-Index reflects the same degree of polarization, we cannot expect the same physiological response from different TIDs emerging in the same Polarization-Index. For example, a Polarization-Index of 2.3 a.u. can result out of 90-3-7 and 50-10-40. Nevertheless, the Polarization-Index seems useful to quantify the level of polarization of TIDs with Z1-percentages between 75 and 95%, which are frequently used in high performance sports (Seiler, 2010).

### Statistical analysis

All statistical procedures were calculated using the statistical package SPSS 21. Average data are expressed as arithmetic mean ± standard deviation, unless otherwise stated. To calculate differences between groups, a *t*-test was employed after testing for normal distribution using a Shapiro–Wilkinson-Test. A paired *t*-test was applied to calculate differences between pre and post-test. An unpaired *t*-test analyzed significant differences between training groups. Cohens d (d_Cohen_) was calculated to estimate effect sizes (Cohen, 1988), defined as follows: trivial: 0–|0.2|, small: |0.2|–|0.6|, moderate |0.6|–|1.2|, large: |1.2|–|2.0|, very large: |2.0|–|4.0|, and infinite: > |4.0| (Hopkins, 2003).

A correlation analysis using Pearson's coefficient identified possible effects of volume spent in the specific training modes, number of days without training, training volume in Z1-Z3, Polarization-Index and changes in endurance variables (i.e., P_2,000 m_, P_2[BLa]_, P_4[BLa]_, $\dot{\text{V}}$O_2max_). Effect sizes of correlation were defined as follows: trivial: 0.0, small: 0.1–0.3, moderate: 0.3–0.5, high: 0.5–0.7, very high: 0.7–0.9, nearly perfect: 0.9, and perfect 1.0 (Hopkins, 2003).

We dichotomized the outcome of the main variable P_2,000 m_ into ΔP_2,000 m_ (≤1.3%; >1.3%) to distinguish between changes smaller or higher than the smallest worthwhile change and applied a Fishers' exact test to calculate if distributions between ΔP_2,000 m_ and Group (PYR; POL), or Polarization-Index (≤2; >2) were different.

## Results

### Training

#### Specific training (boat & rowing ergometer)

The rowing volume and sum of rowing sessions are summarized in Table 2. Rowing training was not significantly different between groups regarding absolute volume, duration or frequency. Small effect sizes indicated a slightly higher distance covered by PYR, while the number of training sessions was slightly higher in POL (Table 2).

**Table 2:** Mean characteristics of the total rowing volume and sum of rowing sessions.

**Variable**	**PYR**	**POL**	***p***	***d*_Cohen_**
Rowing distance (km)	1334 ± 67	1255 ± 264	0.466	−0.41
Rowing duration (min)	5953 ± 315	5919 ± 1216	0.945	−0.04
Sessions (n)	80 ± 4	84 ± 13	0.414	0.42

*PYR, pyramidal training intensity distribution; POL, polarized training intensity distribution*.

#### Training mode

The distribution of rowing (boat and ergometer) and strength training were not significantly different between groups, but there was a tendency for higher volume of *Endurance* and *Other* training in POL with moderate to large effects. Notably, the number of *Endurance* and *Other* sessions were significantly higher in POL compared to PYR, underlined by large effect sizes (Table 3).

**Table 3:** Distribution of training modes during observation period.

**Training mode**	**Duration (min/wk)**				**Percent (%/wk)**				**Sessions (1/wk)**			
	**PYR**	**POL**	***p***	***d*_Cohen_**	**PYR**	**POL**	***p***	***d*_Cohen_**	**PYR**	**POL**	***p***	***d*_Cohen_**
Rowing	541 ± 28	537 ± 110	0.93	−0.05	58 ± 5	54 ± 9	0.35	−0.55	7.2 ± 0.3	7.7 ± 1.2	0.41	0.57
Strength	178 ± 32	149 ± 72	0.36	−0.52	19 ± 3	15 ± 7	0.18	−0.74	1.6 ± 0.4	1.3 ± 0.7	0.39	−0.53
Endurance	144 ± 30	202 ± 68	0.07	1.10	15 ± 3	21 ± 6	0.08	1.27	1.7 ± 0.4	3.0 ± 0.8	0.00	1.37
Other	68 ± 28	100 ± 29	0.06	1.12	7 ± 3	10 ± 3	0.07	1.00	2.2 ± 0.6	3.5 ± 1.2	0.03	1.37
Total	931 ± 50	990 ± 100	0.19	0.75					12.7 ± 0.8	15.5 ± 2.3	0.002	1.63

*PYR, pyramidal training intensity distribution; POL, polarized training intensity distribution; Rowing, boat & rowing ergometer; Strength, resistance training; Endurance, other modes of endurance training than rowing (e.g., spinning, cycling, running); Other, all other kinds of training not mentioned before (e.g., stretching, stabilization training, Yoga)*.

Both groups did not differ regarding the days of illness (*p* = 0.81) amounting to a median of 3 days (min-max: 1–7) in PYR and 4 days (1-11) in POL.

#### Intensity distribution

**Figure 2:**
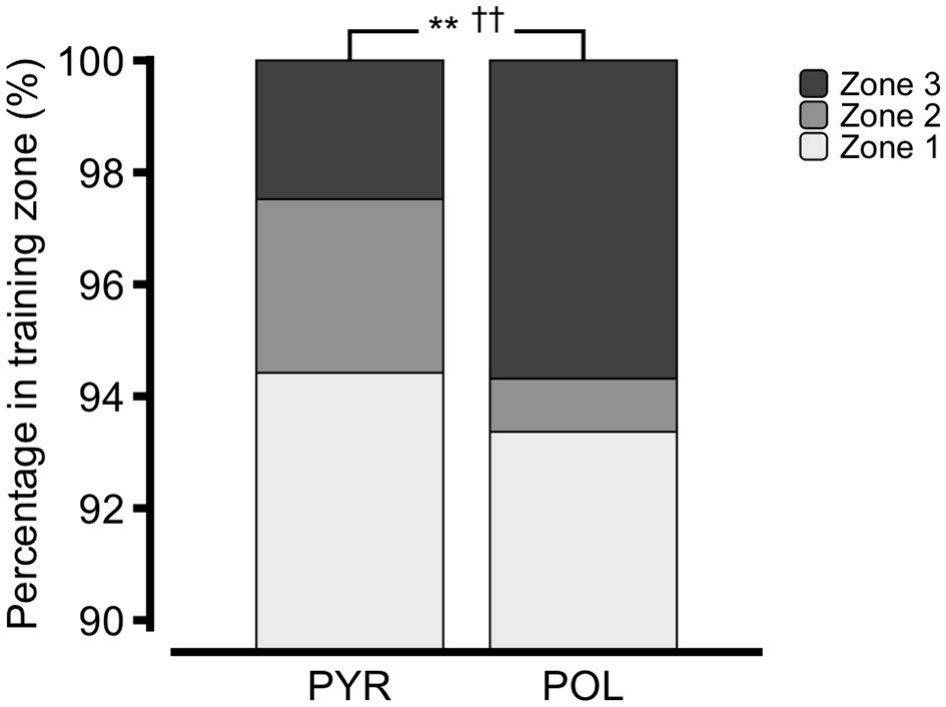
Intensity distribution in boat & ergometer rowing. ^**^ and ^††^: Percentage rowed in Z2 and Z3 differed between pyramidal (PYR) and polarized training intensity distribution (POL) (*p* < 0.01).

Figure 2 shows that percentage of training in Z1 was similar between PYR and POL (94 ± 3% and 93 ± 2%; *p* = 0.37, d_Cohen_ = −0.33), but Z2 was significantly higher in PYR (3 ± 2% and 1 ± 1%; *p* < 0.01; d_Cohen_ = −1.27) and Z3 was significantly higher in POL (2 ± 1% and 6 ± 3%; *p* < 01; d_Cohen_ = 1.79). This emerged into a significantly higher Polarization-Index in POL (1.9 ± 0.4 and 3.0 ± 0.7; *p* < 0.01; d_Cohen_ = 1.93).

**Figure 3:**
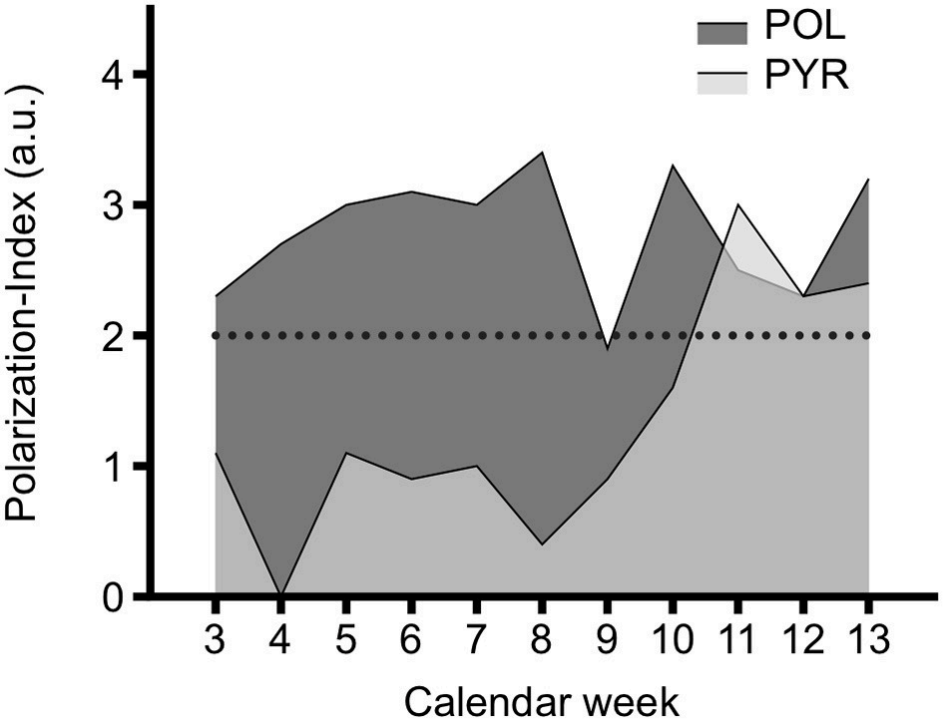
Polarization-Index of a pyramidal (PYR) and a polarized rowing training group (POL) during 11 weeks of intervention and pre-/post-testing. Polarization-Index = log (%Zone 1 × %Zone 3 / %Zone 2). See text for details. Broken line indicates Polarization-Index of 2 a.u., which is defined being the cut off between polarized (> 2) and not polarized (≤2 a.u.) training intensity distribution. The high percentage of Zone 3 in calendar weeks 3 and 13 was due to the pre-/post-testing.

The longitudinal differences in TID of both groups during the 11-wk intervention expressed by means of the Polarization-Index are displayed in Figure 3. The Polarization-Index was significantly higher in POL in calendar week 4, 5, 6, 7, 8, and 10 (*p* = 0.00 to 0.02). No significant differences in the Polarization-Index were found in the other weeks, although with a tendency to be greater in POL (*p* = 0.19 to 0.36) except of weeks 11 and 12. To note, the Polarization-Index clearly increased in PYR toward the end of the study period, starting in calendar week 11 (3.0 a.u.), thereby indicating a considerable increase of Z3-intensities.

### Performance

In the complete sample, P_2,000 m_ increased significantly (*p* = 0.03) from 443 ± 30 W to 449 ± 26 W, corresponding to an improvement in 2,000 m time of ~2 s (pre: 370.1 ± 8.7 s to post: 368.2 ± 7.2 s, representing a small effect (d_Cohen_ = −0.24). Average changes between PYR and POL or within PYR and POL from pre to post-test were trivial or small and not significant (Table 4).

**Table 4:** Changes in performance after 11 weeks of training in national elite rowers.

**Variable**	**PYR**						**POL**						**PYR vs. POL**	
	**Pre**	**Post**	**Δ%**	***n***	***p***	***d*_Cohen_**	**Pre**	**Post**	**Δ%**	***n***	***p***	***d*_Cohen_**	***p***	***d*_Cohen_**
$\dot{\text{V}}$O_2max_ (mL/min/kg)	64 ± 3	64 ± 2	1.7 ± 5.6	6	0.522	0.00	68 ± 7	68 ± 7	0.6 ± 2.8	6	0.686	0.00	0.712	0.22
Duration 2,000 m test (s)	368.8 ± 7.6	367.0 ± 6.4	−0.5 ± 0.6	7	0.060	−0.26	372.0 ± 10	369.8 ± 8.4	−0.5 ± 0.9	6	0.221	−0.24	0.962	0.03
P_2000_m (W)	447 ± 27	454 ± 24	1.5 ± 1.7	7	0.057	0.27	438 ± 36	444 ± 30	1.5 ± 2.6	6	0.258	0.00	0.916	0.06
P_2[BLa]_ (W)	291 ± 26	298 ± 19	3.0 ± 5.6	7	0.232	0.31	297 ± 16	297 ± 27	0.2 ± 5.9	7	0.897	0.00	0.441	0.42
P_4[BLa]_ (W)	336 ± 25	341 ± 20	1.9 ± 4.8	7	0.369	0.22	337 ± 17	336 ± 24	−0.5 ± 4.1	7	0.770	−0.05	0.369	0.50

*PYR, Pyramidal training intensity distribution; POL, polarized training intensity distribution; $\dot{\text{V}}$O_2max_, maximal oxygen uptake; P_2,000_m, average power in 2,000 m rowing ergometer test; P_2[BLa]_ and P_4[BLa]_, Power output with [blood lactate] 2 and 4 mmol/L. PYR vs. POL was calculated from the absolute difference between pre- and post-test of each group*.

$\dot{\text{V}}$O_2max_, P_2[BLa]_, P_4[BLa]_ did not significantly change from pre to post or between groups. A small increase of average P_2[BLa]_ and P_4[BLa]_ was detected in PYR only, where three rowers (43%) improved P_4[BLa]_ by more than 3.3% in contrast to POL with only one rower (14%) who improved above this threshold. The unbalanced improvement in P_2[BLa]_ and P_4[BLa]_ between groups is also reflected by small effect sizes in PYR and trivial effect sizes in POL (Table 4).

**Figure 4:**
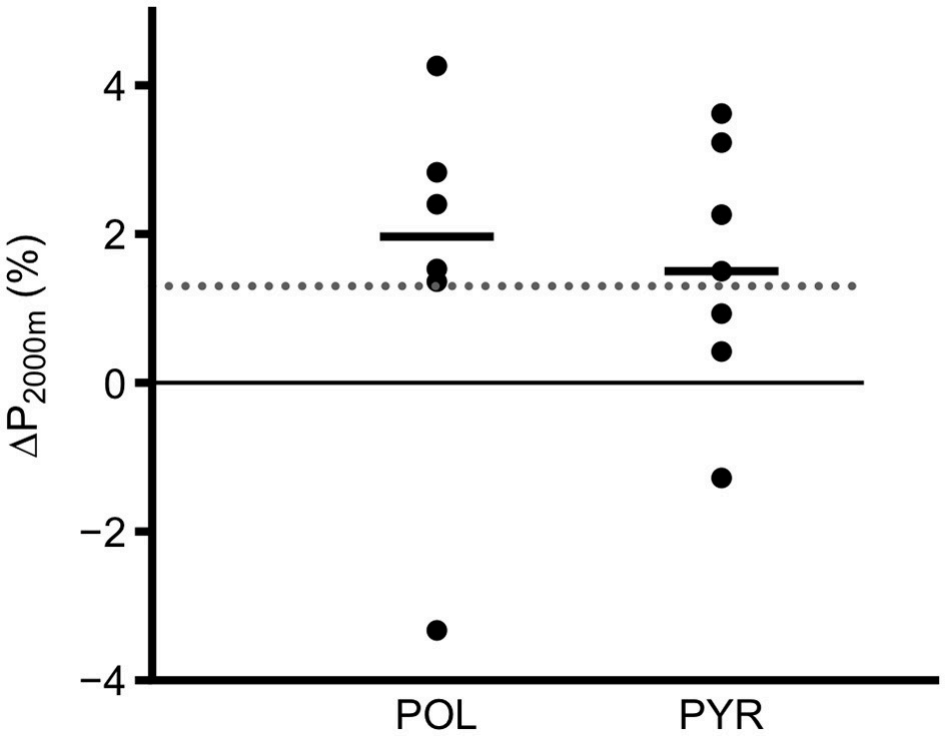
Individual change of average power in 2,000 m rowing-ergometer test (P_2,000 m_) after 11-weeks between rowers following a polarized (POL) or pyramidal (PYR) training intensity distribution. The dashed line corresponds to estimated error of measurement for P_2,000 m_ on Concept 2 rowing ergometers of 1.3% according to Soper and Hume (2004). Short lines indicate median of each group.

On an individual level five out of six (83%) rowers with POL improved P_2,000 m_ above the estimated error of measurement of 1.3% (95%CI 0.9-2-9; Soper and Hume, 2004). In PYR, four out of seven (57%) rowers improved P_2,000 m_ above 1.3% (Figure 4).

### Training and performance: correlation analysis

Correlation analysis indicated, that changes in P_2,000 m_ became smaller with greater volume of *Other* training like e.g., stretching (*r* = −0.61; *p* = 0.03).

Changes in performance variables (P_2[BLa]_, P_4[BLa]_, P_2,000 m_, $\dot{\text{V}}$O_2max_, and duration in 2,000 m ergometer test) were not significantly correlated to total days without training (highest absolute *r* = 0.11 with *p* = 0.71), neither health related (highest absolute *r* = 0.42; *p* = 0.18) nor related to scheduled days off (highest absolute *r* = 0.26; *p* = 0.41).

A high correlation was found between changes in $\dot{\text{V}}$O_2max_ and absolute weekly volume (*r* = 0.58; *p* = 0.05) or percentage (*r* = 0.59; *p* = 0.04) spent in Z2. However, average changes in $\dot{\text{V}}$O_2max_ were small, amounting to 0.1 L/min which is within the technical error of measurement of 0.03–0.21 L/min (95% confidence interval) for the device used in this study (Larsson et al., 2004).

Negative correlation coefficients indicated, that the higher the absolute volume of Z3-training, the smaller the increase in P_2[BLa]_ (*r* = −0.56; *p* = 0.02) and P_4[BLa]_ (*r* = −0.53; *p* = 0.05). Similar results were obtained for percentage of Z3 and P_2[BLa]_ (*r* = 0.63; *p* = 0.02) and P_4[BLa]_ (*r* = −0.59; *p* = 0.03). In line with the previous result, smaller changes in P_2[BLa]_ (*r* = −0.58; *p* = 0.03) and P_4[BLa]_ (*r* = −0.64; *p* = 0.01) were correlated with higher Polarization-Index.

### Distribution of worthwhile change in 2,000 m ergometer test, group, and polarization-index

Even though more rowers in the POL-group increased P_2,000 m_ (Figure 4, Table 4) the distribution between groups regarding the dichotomized variable change ≥1.3% vs. change <1.3% was not different (Fisher's exact: *p* = 0.56), basically due to an outlier with ΔP_2,000 m_ of −3.3% (Figure 4).

**Figure 5:**
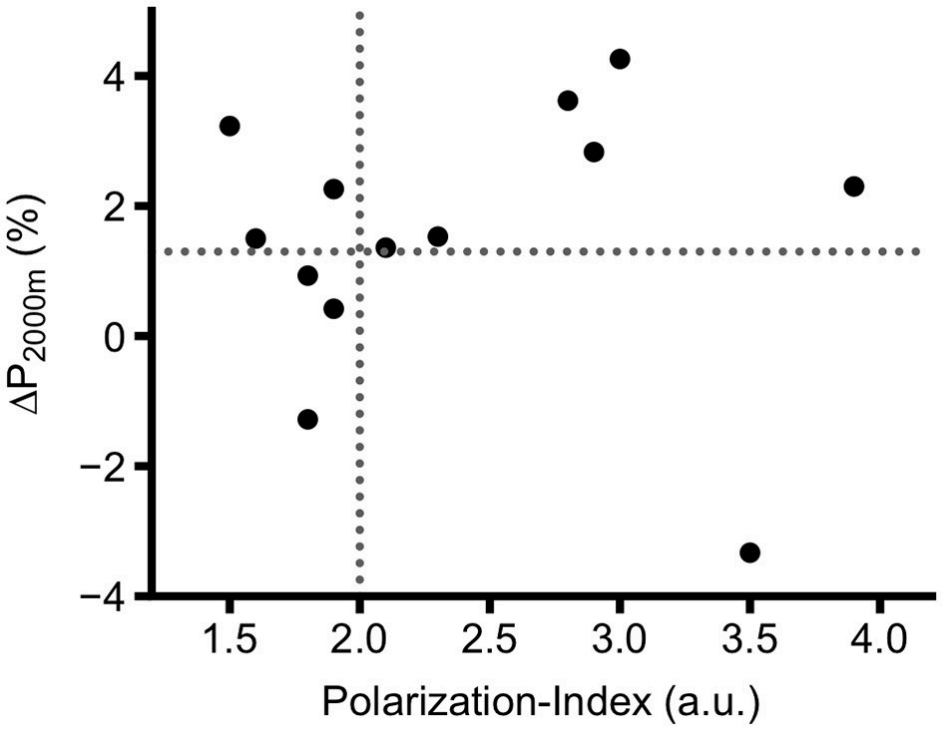
Individual change (%) of average power in 2,000 m rowing ergometer test (P_2,000 m_) in 14 highly trained competitive U23 rowers. Horizontal dashed line indicates Polarization-Index of 2, which is defined being the cut off for polarized training intensity distribution (see text for details). Vertical dashed line indicates 1.3%, being the estimated error of measurement for P_2,000 m_ according to Soper and Hume (2004).

Figure 5 indicates that—irrespective of the group allocation—six out of seven (86%) rowers with a Polarization-Index >2 increased P_2,000 m_ more than 1.3%. In contrast, only three out of six rowers (50%) with a Polarization-Index ≤2 improved their P_2,000 m_ above the smallest worthwhile change. However, distribution between the dichotomized variable *Polarization-Index* (≤2.0; >2.0) vs. Δ*P*_2,000 m_ (≤1.3%; >1.3%) was not significantly different (Fishers exact test: *p* = 0.27).

## Discussion

We compared two TIDs (POL vs. real-life PYR) in national elite rowers with increasing percentage of Z3-training during the last 3 weeks of a 11-wk training period. Notably, percentage of Z1 was clamped to ~93% in both groups. In summary, POL did not show to be significantly superior to PYR regarding any physiological determinant of rowing performance, but small effects indicated improved P_2[BLa]_ and P_4[BLa]_ in PYR only. However, irrespective of the groups, we observed a higher percentage of worthwhile improvements regarding P_2,000 m_, which is the key variable in rowing, when the Polarization-Index was ≥2.1 a.u.

On a group level both TID models allowed for improvements in P_2,000 m_, which is the most reliable and accepted surrogate measure of rowing performance (Hahn et al., 2000; Mäestu et al., 2005). Also Ingham et al. who compared POL to a low-intensity group, did not find POL to be superior in rowers, but in contrast to our results, the maximal oxygen uptake increased in both of their groups probably attributable to the much lower training status of those club rowers who were 13% slower in the 2000 m ergometer test compared to our athletes (Ingham et al., 2008). The athletes in our study were highly trained rowers including several medalists of Junior and U23 world championships, and it is well-known that significant and worthwhile improvements in maximal oxygen uptake are not easily achieved in this group of athletes.

Data from other endurance athletes (e.g., cyclists and runners) suggested, that POL might be superior regarding key endurance variables including $\dot{\text{V}}\text{O}$_2max_, P_2[BLa]_, P_4[BLa]_, or time-trial performance (Neal et al., 2013; Stöggl and Sperlich, 2014) and confirmed by several uncontrolled training studies using observational data (Billat et al., 2001; Seiler and Kjerland, 2006; Sandbakk et al., 2011; Tønnessen et al., 2014) and also studies emphasizing high-intensity in rowers (Ní Chéilleachair et al., 2016).

We will therefore briefly discuss possible reasons why there were no clear differences in important physiological determinants between POL and PYR, including illness, training volume, mode, and TID.

### Illness

Due to the typical rough weather conditions between January and March in central Europe, the pre-competition period of the rowers in our study was frequently disturbed by minor illnesses like colds and upper respiratory tract infections. Several athletes in our group experienced minor illnesses with the cancellations of single and multiple training sessions. However, based on the training diaries and our statistical analysis we did not detect any significant correlation between days of illness and performance outcome or any differences between groups. As the athletes may not have always reported re-scheduling of training due to minor illness (e.g., substituting cycle ergometer for rowing) it may be possible that mild infectious diseases have affected the outcome of the study. However, it is noteworthy that frequent re-scheduling is a real-life circumstance within the training process thereby constantly altering the prescribed TID.

### Training volume

Data concerning the precise training volumes of high performance rowers are scarce. The average weekly volumes of groups and single cases vary from 102 km/wk (Seiler and Tønnessen, 2009), 111.9 ± 43.7 km/wk (Tran et al., 2013), 119 km/wk (Lacour et al., 2009), 124 km/wk (Mikulic, 2011), 127 km/wk (Bourgois et al., 2013) to 135 km/wk (Nybo et al., 2014). Based on the aforementioned reports, the average training volume of elite rowers amounts to ~120 km/week. In our study rowing training averaged 114 km/wk (POL) to 121 km/wk (PYR) which appears to be relatively high since most of our rowers were U23-rowers, who generally train less then world-class rowers of higher age, but reasonably more than juniors who row ~97.1 ± 19.5 km/wk (Guellich et al., 2009). We therefore assume, that training volume in our study was *per-se* high enough to allow for changes in performance, independent of alterations in TID.

Volume of *Endurance* and *Other* training was moderately higher in POL. The number of the according sessions was significantly higher in POL with even large effect sizes (Table 3). While the high volume of non-specific endurance training was without negative effects, *Other* training (e.g., stretching) was obviously not effective above a certain threshold, as indicated by the negative correlation with P_2,000 m_.

### Duration of the intervention

The intervention period of 11 weeks is generally long enough to allow for physiological adaptations and is comparable to studies in elite (Stöggl and Sperlich, 2014) or sub-elite athletes (Neal et al., 2013).

### Training intensity distribution

In our study the percentage of Z2 and Z3 was significantly different between PYR and POL with large effect sizes, indicating a relevant difference between groups in intensities near and above lactate threshold. However, the accumulated percentage of training in Z2 and Z3 did not exceed 7% in any group, which is very similar to classical rowing data (Steinacker et al., 1998; Guellich et al., 2009) but appears to be relatively low compared to the majority of current data in rowers varying between 7% (Bourgois et al., 2013), 15% (Nybo et al., 2014), 19% (Seiler and Tønnessen, 2009), and 23% (Plews et al., 2014), and as well as studies investigating POL involving other disciplines [20% (Neal et al., 2013) and 32% (Stöggl and Sperlich, 2014)]. Thus, we assume that the equally low percentage spent in Z2 and Z3 was not a sufficient stimulus to improve e.g., oxygen uptake, since high-intensity exercise is more effective in inducing central adaptations, as reported in highly trained cyclists (Laursen et al., 2002). In addition, our study clearly indicates that a polarized TID is not superior as such, but necessitates an optimal and probably higher sum of Z2 and Z3 intensities than realized by our POL-group. Obviously, POL requires an optimal and probably greater overall proportion of Z2- and Z3-intensitiy in contrast to the TID accomplished by our POL-group.

As recommend by the scientific literature (Bangsbo et al., 2010; Tønnessen et al., 2014) our study involved an increase in intensity over time especially in PYR to taper for the first national trials. Since especially PYR increased Z3 toward the end of the study, which is a real-training procedure, and since greater amounts of Z3 are reported to introduce rapid adaptions (Driller et al., 2009; Ní Chéilleachair et al., 2016), the great amount of Z3 in PYR during the last 2 weeks of the study period suggests, that pronounced and short periods of polarized training after several weeks of Z1 and Z2 training are a sufficient stimulus to improve performance. We assume, this effect of real-life TID very likely contributed to the lack of differences between POL and PYR.

Due to the aforementioned reasons including illness, fatigue or environmental conditions, some rowers in POL and PYR showed a greater “polarization” than others, as expressed by the Polarization-Index. Interestingly, we found more frequent improvements of P_2,000 m_ in those rowers who trained more polarized, irrespective of the group they were allocated to. Judging from the plot in Figure 5, we observed (with the exception of one outlier) that higher levels of polarization led to an increase of more than 1.3% (i.e., the estimated error of measurement) in P_2,000 m_, especially if Polarization-Index was >2.3 a.u. The notion, that variation including polarization needs to be consequently implemented to offer considerable advantages is plausible, because other studies reported increases of performance and/or $\dot{\text{V}}\text{O}$_2max_ in rowers after high intensity-interventions between four (Driller et al., 2009) and eight weeks (Ní Chéilleachair et al., 2016). Since the average changes in P_2,000 m_ were not related to any physiological variable, other factors including efficiency and pacing may have accounted for the changes.

P_4[BLa]_ is an established and valuable parameter to assess performance in rowing (Ingham et al., 2002; Smith and Hopkins, 2012) and a relevant fitness marker for many high performance coaches in rowing (Altenburg et al., 2012). Surprisingly, we did not find increases of P_2[BLa]_ and P_4[BLa]_ in POL, but small and more frequent improvements of P_4[BLa]_ in PYR. This is in line with others, also reporting minor improvements in P_4[BLa]_ with POL compared to a control group emphasizing low-intensity (Ingham et al., 2008).

According to our data, the higher percentage of Z3 (or the lower percentage of Z2) in POL contributes to the unaltered or even lowered P4_[BLa]_ values in POL, as indicated by the high negative correlation between P4_[BLa]_ and Polarization-Index or percentage of Z3. The notion, that Z2 training is essential for improvements in rowing performance is in line with other reports on successful elite rowers, whose training schedule always incorporated higher percentage of Z2-training, as indicated by data from New Zealand (Plews et al., 2014), Norway (Seiler and Tønnessen, 2009), and Denmark (Nybo et al., 2014). In addition, coaches of the POL-group reported (but did not quantify) that during the intervention period, the speed in Z1 was partially lower than before the study, to allow for recovery from fatigue inducing Z3 sessions. Since energy expenditure increases with boat speed by 2.2- to 2.4-fold power (Secher, 1993), we assume that the sessions at the lower end of the T1-range with low metabolic cost and muscular force were insufficient to further stimulate adaptions, thereby explaining the lack of improvement in POL. Differences of TID within Z1 are probably relevant to induce further adaptations, however, since we did not collect the necessary data, this notion warrants further investigation.

### Limitations

Coaches and athletes volunteered to participate in the study and were fully informed about the two training regimes, but the scattering of the TIDs within the POL group indicates that some rowers did not entirely follow the training program in the same consequence. This limitation of our study was attributable to concerns by coaches and athletes to adopt a new TID with the possibility for non-functional overreaching. Further, minor illnesses and environmental factors caused elimination or altering of sessions with higher intensity, which might not have been reported in the diaries. In addition, the athletes in our study rowed in different boat types, ranging from single sculls to crew boats like four and quadruple sculls. Rowing in crew boats hinders the strict individual adherence to a prescribed TID, because the individual rower has to adapt to a given pace that emerges from the skills and physiological capacity of the crew, which also contributed to the scattering of individual TIDs and physiological changes, furthermore partly explaining the differences to studies in e.g., cyclists. Another limitation of our study is that the calculation of the TIDs is not based on HR-logfiles, but on the rowers' diary entries, based on their HR-measurements, time, and distance trained in each zone. The lack of HR-logfiles for direct analysis hindered us to distinguish between training intensities within a given intensity zone. Nevertheless, we cross-checked the entries with the coaches and associated rowers to overcome this limitation. An additional measurement after 8 weeks of the study would have theoretically allowed to quantify the impact of the high-intensity training in PYR during the last three weeks of the study (Figure 3). However, the interruption of the training process due to several days of obligatory tapering before the measurements and two additional days of testing would have been an unrealistic training scenario, thereby causing another limitation and moreover not acceptable by the coaches and athletes. Finally, athletes were inevitably aware of the group (POL or PYR) they were allocated to. We therefore cannot exclude expectancy effects.

## Conclusion

We conclude that on a group level, POL is not superior to a dynamic, real-life PYR distribution when percentage of Z1 is clamped to ~93%. However, it seems POL can have ergogenic effects, i.e., improved P_2,000 m_, if applied consistently and with a Polarization-Index ≥2.3 a.u. Taking previous data of elite non-rowing athletes into account, we assume that higher percentage of Z3 is necessary to achieve potential ergogenic superiority of POL compared to PYR.

## Ethics statement

This study was carried out in accordance with the recommendations of ethical review board of the University of Ulm with written informed consent from all subjects. All subjects gave written informed consent in accordance with the Declaration of Helsinki. The protocol was approved by ethical review board of the University of Ulm.

## Author contributions

GT: Planned and designed the study, conducted measurements, analyzed the data, prepared the manuscript. KW: Planned and designed the study, conducted measurements, analyzed the data, edited the manuscript. MS: Conducted measurements, edited the manuscript. JS: Designed the study, edited the manuscript. MB: Analyzed the data. BS: Designed the study, analyzed the data, edited the manuscript.

### Conflict of interest statement

The authors declare that the research was conducted in the absence of any commercial or financial relationships that could be construed as a potential conflict of interest.
